# Emphysematous Osteomyelitis: Three Rare Cases

**DOI:** 10.7759/cureus.103435

**Published:** 2026-02-11

**Authors:** Zachary C Culley, Samantha M Harrington, Nicholas R Hauser, Justin R Samanta, Ramy A Shoela

**Affiliations:** 1 Radiology, Saint Louis University School of Medicine, St. Louis, USA; 2 Radiology, Saint Louis University Hospital, St. Louis, USA

**Keywords:** anaerobic bacteria, bone necrosis, clinical presentation of emphysematous osteomyelitis, ct in osteomyelitis, emphysematous osteomyelitis, gas-producing infections, mri in osteomyelitis, necrotizing soft tissue infections (nsti), radiologic imaging in osteomyelitis, sepsis and osteomyelitis

## Abstract

Emphysematous osteomyelitis is a rare condition caused by gas-producing bacteria in the bone and may be visualized by the presence of intraosseous gas on imaging. We report three cases of emphysematous osteomyelitis. The first case is an 80-year-old with acute myeloid leukemia with emphysematous osteomyelitis of the sacrum, right iliac bone, and multiple vertebrae and ribs. Blood cultures grew *Enterococcus faecium* and *Clostridium* spp. The second case is a 45-year-old man with diabetes mellitus with emphysematous osteomyelitis of the proximal left tibia with blood cultures positive for *Proteus mirabilis* and *Clostridium perfringens*. The final case is a 57-year-old male with diabetes mellitus who presents with emphysematous osteomyelitis of the left fifth metatarsal head. Wound cultures were positive for *Klebsiella pneumoniae* and *Proteus mirabilis*. Emphysematous osteomyelitis is associated with significant morbidity and mortality, and so an accurate and quick diagnosis is important for the radiologist to make.

## Introduction

Emphysematous osteomyelitis (EO) is a rare condition in which gas-producing bacteria (e.g., *Pseudomonas*, *Enterococcus*, *Escherichia coli*, *Salmonella*, *Staphylococcus aureus*, *Clostridium* spp., *Klebsiella*) infiltrate bone tissue. The true incidence rate of EO is unknown, but it is considered rare, with the largest reviews ranging from 25 to 45 cases worldwide [1]. Patients with EO are mostly older adults (age 60-75) and are primarily men (80%) [1]. Risk factors include diabetes mellitus (DM), malignancy, alcohol abuse, Crohn's disease, and other etiologies causing immunosuppression [2,3]. The condition is potentially fatal if not diagnosed and treated rapidly [4]. The mortality rate of EO is estimated to be 24-32% [1].

All the listed patients in this report had comorbidities regularly seen with this condition, such as malignancy, diabetes, and a prolonged history of alcohol/smoking. While recognizing these signs is important for EO to be a part of a differential diagnosis, imaging with a CT followed by an MRI is crucial for a complete diagnosis of this disease. Intra-osseous gas is better visualized on CT, while the extent of bone infection can be better discerned with MRI. Findings of intra-osseous gas on imaging in conjunction with a bone biopsy remain the gold standard for diagnosis, although blood cultures may be diagnostic if positive in the context of convincing imaging findings and clinical context [5]. If multiple sites of EO are seen on imaging, a full-body screening is recommended to ensure that no additional sites of infection are missed. We report three unique cases of EO, the first of which demonstrated multifocal EO via bloodstream seeding, a rare infection that has only been documented a few other times [6,7].

## Case presentation

Case 1

An 80-year-old Caucasian male with a past medical history significant for acute myeloid leukemia (AML) presented to the Emergency Department (ED) as a transfer from an outside hospital. He presented to the outside hospital with worsening generalized weakness and shortness of breath. His medication regimen prior to hospitalization included venetoclax (Venclexta) 100 mg PO BID for treatment of AML in addition to opportunistic infection prophylaxis, including fluconazole (Diflucan) 200 mg PO QD, levofloxacin (Levaquin) 500 mg PO QD, and valacyclovir (Valtrex) 500 mg PO BID. He smoked 1.5 packs/day of cigarettes.

Laboratory results collected at the outside hospital revealed pancytopenia, elevated CRP, and elevated liver enzymes (Table 1). Blood cultures were found to be positive for *Clostridium* spp. and *Enterococcus faecium*. 

**Table 1:** Laboratory investigation of case 1.

Laboratory Name	Laboratory Value	Laboratory Reference Range
WBC	200 cells/µL	3,500-10,500 cells/µL
Hgb	7.8 g/dL	12.0-17.6 g/dL
HCT	23.6%	35.2-51.7%
Platelet count	15,000 cells/µL	150,000-400,000 cells/µL
Neutrophil % manual	40%	44-73%
Lymphocytes % manual	44%	20-43%
CRP	32.88 mg/dL	≤0.5 mg/dL
Lactic acid	2.7 mmol/L	0.5-2.2 mmol/L
ALP	811 U/L	38-126 U/L
ALT	42 U/L	0-61 U/L
AST	93 U/L	5-40 U/L
Albumin	2.9 g/dL	3.4-5.0 g/dL

**Figure 1:**
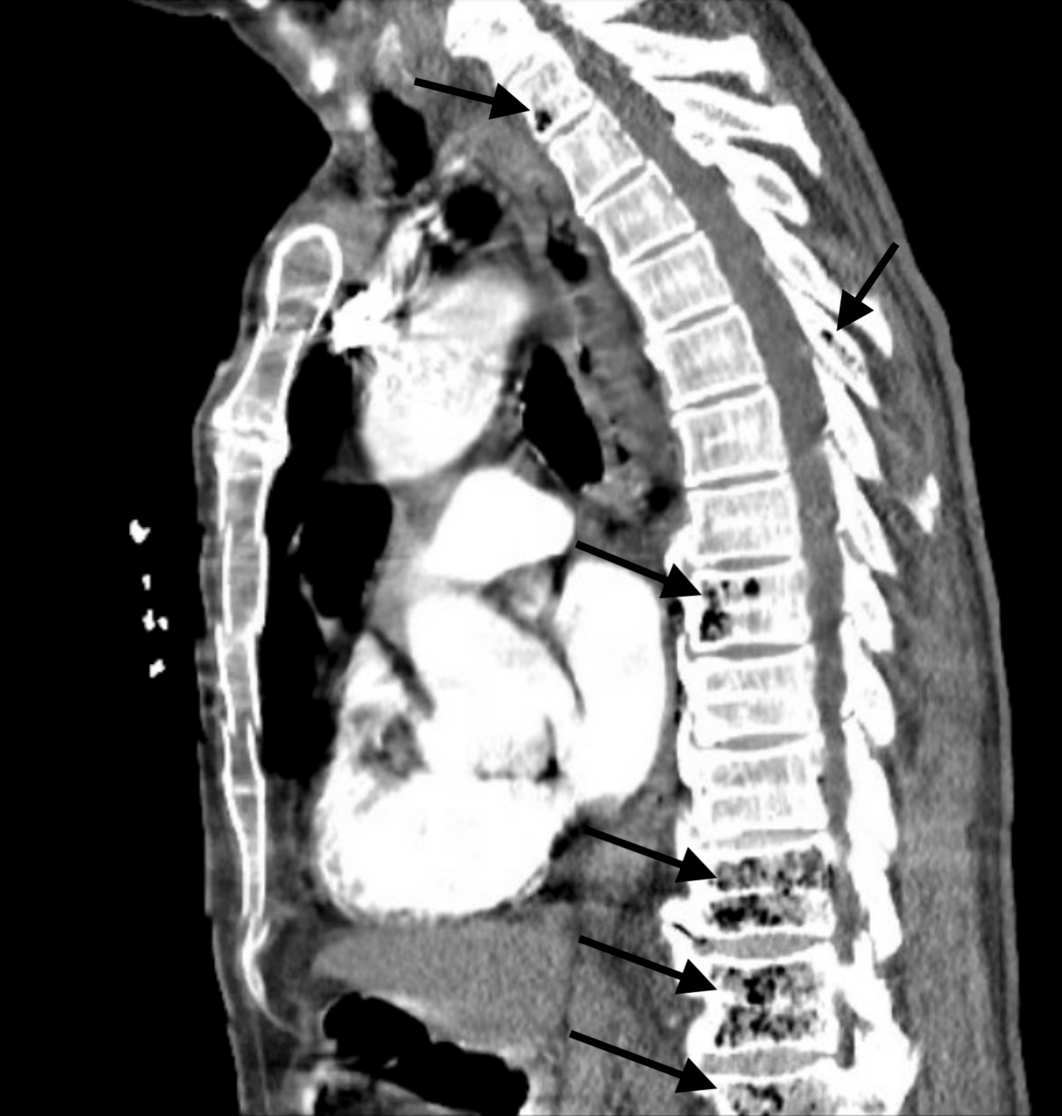
Emphysematous osteomyelitis of the thoracic and lumbar spine on CT. Sagittal CT angio chest in an 80-year-old Caucasian man presenting with worsening generalized weakness, dyspnea, and tachycardia with black arrows showing diffuse intraosseous gas within T1, T8, T11, T12, and L1 vertebrae as well as the T5 spinous process.

CT angiography of the chest with contrast performed at the outside hospital was unremarkable for pulmonary embolism; however, diffuse intraosseous gas was incidentally found, most notably within the thoracic/visualized lumbar vertebral levels T1, T8, T11, T12, and L1 vertebrae as well as the T5 spinous process with extension into the posterior mediastinum and spinal canal (Figure 1).

At the outside hospital, he was given IV vancomycin 1750 mg × 1 dose and IV cefepime 2000 mg q8h × 2 doses, and then was switched to IV meropenem 1000 mg q6h × 2 doses before his transfer 24 hours later. 

Upon transfer from the outside hospital, the patient's vital signs were as follows: temperature 37.1℃, pulse rate 121 bpm, respiratory rate 22, and blood pressure 126/76. The initial oxyhemoglobin saturation (SpO2) was 100% on room air by pulse oximetry. The patient's BMI was 19.48 kg/m². 

Physical exam revealed a cachectic appearance, dry mucous membranes, tachycardia, and coarse breath sounds bilaterally. Blood cultures taken on hospital day 0 were positive for *Enterococcus faecium*, initially susceptible to ampicillin and vancomycin, and later found on hospital day 3 to be resistant to ampicillin. 

The patient was placed on IV vancomycin 1250 mg daily, piperacillin-tazobactam 3.375 g q8h, IV micafungin 100 mg q24h, and oral valacyclovir 500 mg BID. Venetoclax was held. One dose of daptomycin 675 mg was given on hospital day 2 and then discontinued as *Enterococcus* was initially ampicillin susceptible.

**Figure 2:**
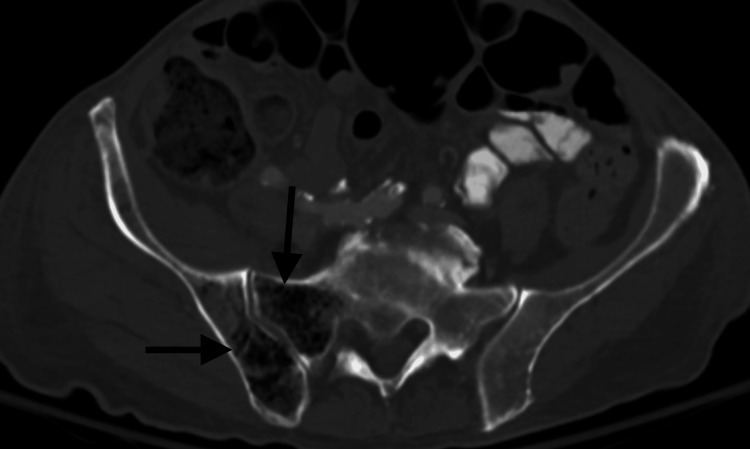
Emphysematous osteomyelitis of the sacrum and pelvis. Axial CT abdomen/pelvis in an 80-year-old Caucasian man presenting with worsening generalized weakness, dyspnea, and tachycardia with black arrows demonstrating emphysematous osteomyelitis of the right iliac bone and right sacroiliac joint.

On hospital day 1, a CT of the abdomen and pelvis with contrast showed findings consistent with a perforated duodenal wall. Interosseous gas was present in the sacrum and right iliac bone as well (Figure 2).

**Figure 3:**
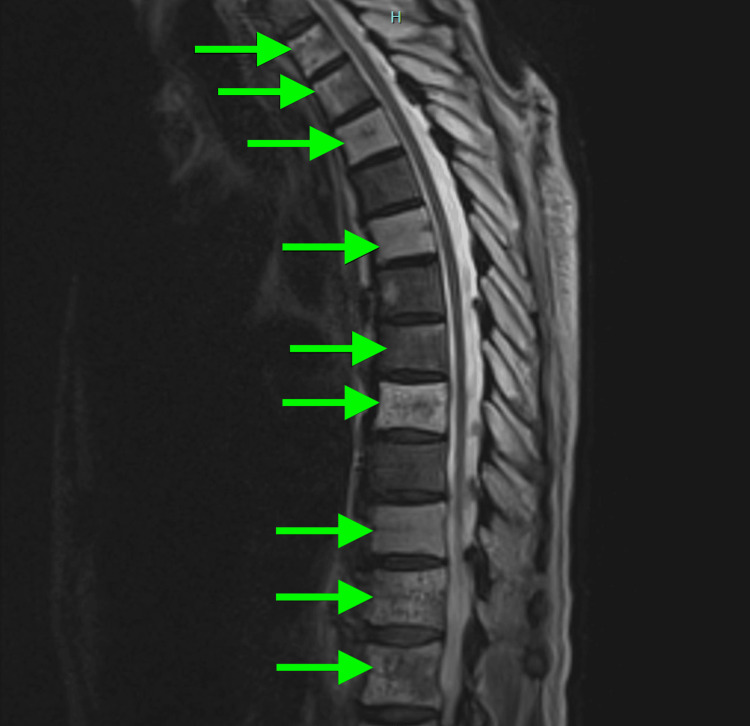
Emphysematous osteomyelitis of the thoracic spine on MRI. Sagittal T1-weighted MRI of the thoracic spine in an 80-year-old Caucasian man presenting with worsening generalized weakness, dyspnea, and tachycardia, showing heterogeneous appearance of the bone marrow in multiple vertebral bodies, including T1, T2, T3, T5, T7, T8, T10, T11, and T12, concerning for emphysematous osteomyelitis.

On hospital day 3, an MRI of the thoracic and lumbar spine was performed. The MRI demonstrated a markedly heterogeneous appearance of the bone marrow in multiple vertebral bodies, including T1, T2, T3, T5, T7, T8, T10, T11, T12, L1, as well as the right sacrum and right iliac wing, all concerning for emphysematous osteomyelitis (Figure 3).

Interventional radiology was consulted for a possible bone biopsy of a vertebral body, but it was deemed low-yield, given that the risks likely outweighed the benefits. Therefore, the bone biopsy was not performed. 

Despite continued antibiotics and supportive care, his condition continued to deteriorate, and he was offered palliative care before his passing on hospital day 15. 

Case 2

A 45-year-old man with a past medical history of asthma, DM, hyperlipidemia (HLD), hypertension (HTN), tobacco use, and alcoholic cirrhosis presented to the ED as a transfer with concern for left knee septic arthritis and a posterior knee abscess after experiencing left knee pain for the last several months. His knee pain worsened with movement and improved with rest. The patient was ambulatory on initial evaluation. 

He was recently treated for sigmoid diverticulitis at an outside hospital, for which he was given antibiotics (piperacillin-tazobactam, dosage unknown), prednisone, and then was discharged. A few days later, he returned to the hospital with bacteremia, and blood cultures were positive for *Proteus mirabilis* and *Clostridium perfringens*. The patient was given amoxicillin-clavulanate and metronidazole and subsequently discharged. He was again readmitted to the hospital a month later before getting transferred for higher level care. 

On physical exam, the skin of the left lower extremity was intact and without lesions. The leg was non-tender to palpation and tender to passive range of motion (ROM) at the level of the left knee joint. Movement and sensation of the left lower extremity were intact. Left dorsalis pedis and posterior tibial pulses were 2+. 

Initial laboratory results demonstrated leukocytosis (WBC 13,200 cells/µL), low hemoglobin (10.1 g/dL), and elevated inflammatory markers such as C-reactive protein level (4.4 mg/dL), erythrocyte sedimentation rate (97 mm/hour), and lactic acid (3.0 mmol/L). His LRINEC score was 2.

Upon initial imaging, an X-ray of the left tibia/femur/knee demonstrated avascular necrosis of the femoral head with moderate narrowing of the lateral knee compartment and a small joint effusion with soft tissue swelling of the left knee. No fracture or dislocation was identified. Orthopedics and acute care surgery were consulted, for which orthopedics performed an arthrocentesis. The patient was admitted to the hospital, and empiric antibiotics were initiated following this regimen: IV clindamycin 0.9 g q8h, IV cefepime 2 g q8h, and IV vancomycin 1.5 g q8h. 

On admission to the hospital, irrigation and surgical debridement of the left knee were performed due to concern for septic arthritis of the left knee. During this procedure, a left posterior knee abscess was visible, and copious purulent drainage was noted. Cultures of the synovial fluid and the left posterior knee abscess were sent. No organisms were noted upon culture gram and stain, anaerobic culture, or fungal culture of the posterior knee abscess. Synovial fluid culture workup was negative as well. Synovial fluid analysis demonstrated red colored fluid with bloody clarity. The WBC calculation was elevated (43,707/µL (N = 0-200/µL)) with 98% segmented neutrophils, 1% lymphocytes, and 1% monocytes. Red blood cell calculated amount was 128,150/µL (no reference range provided by institution). Pathologic examination of the synovial fluid demonstrated neutrophilic predominance. Blood cultures × 2 were performed and were negative. Urine chlamydia and gonorrhea amplified probe was negative. 

After surgical debridement, IV clindamycin was switched to PO metronidazole 500 mg q8h after 24 hours of inpatient treatment per infectious disease recommendations. Cefepime was continued for 6 days duration. Vancomycin dosages were gradually decreased to IV vancomycin 1 g q8h over a 13-day duration, averaging a 0.25 g decrease in dosage every four days. 

**Figure 4:**
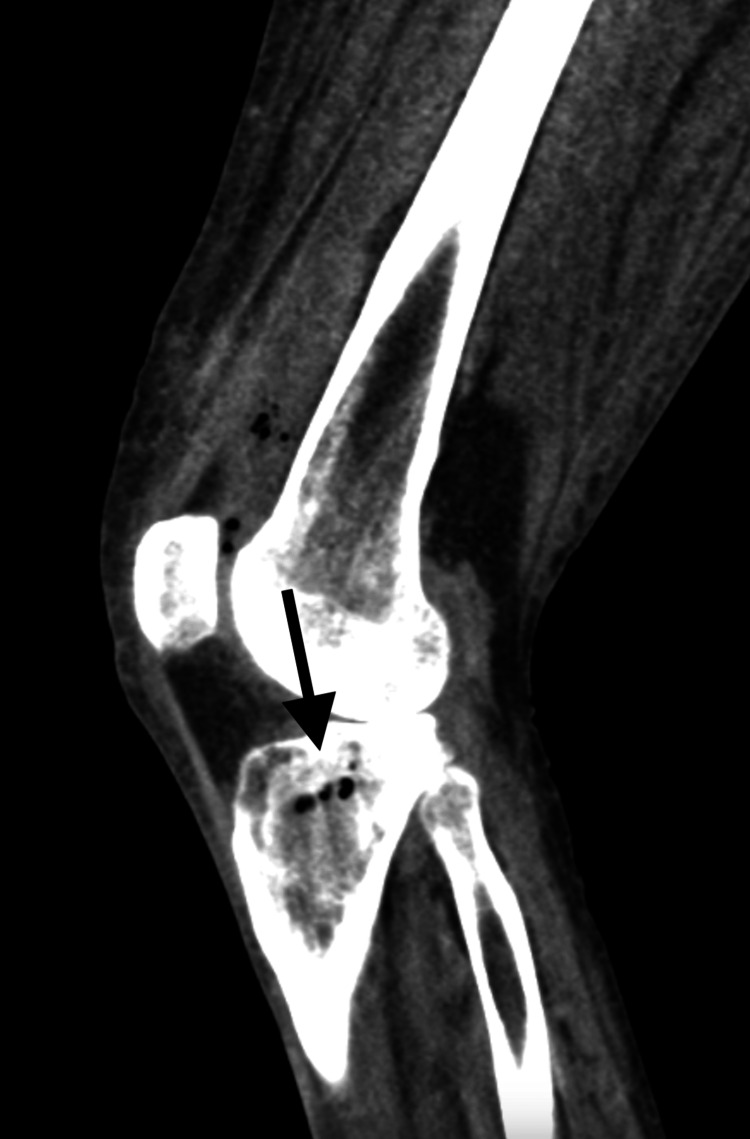
Emphysematous osteomyelitis of the left proximal tibia. Sagittal CT of the left knee joint in a 45-year-old man with knee pain reveals emphysematous osteomyelitis of the left proximal tibia (black arrow).

On hospital day 6, a CT angiography of the lower left extremity revealed septic arthritis, synovitis, and EO of the proximal left tibia with locules of gas seen in the medullary cavity (Figure 4). On hospital day 7, the patient developed a fever to 100.9°F. A repeat CT of the left femur demonstrated slightly increased left knee effusion containing gas, continued intraosseous tibial gas, continued femoral head avascular necrosis, and continued diffuse subcutaneous edema. 

On hospital day 8, a repeat incision and drainage (I&D) with wound vacuum placement was conducted. Infectious disease recommended starting IV piperacillin-tazobactam 3.375 g in 0.9% NaCl q8h and restarting IV clindamycin 900 mg q8h. Clindamycin was continued for eight days. On hospital day 9, the patient developed an acute kidney injury (AKI) that worsened despite intravenous fluid therapy. Nephrology was consulted. On hospital day 12, a repeat washout of the left knee was conducted. Repeat cultures were performed following these I&Ds, and fungal, anaerobic, acid-fast bacilli, and culture gram and stain were negative. 

On hospital day 18, the patient developed worsening mental status, and a CTH was obtained, which revealed no acute intracranial process. The patient was diagnosed with acute encephalopathy likely secondary to gabapentin toxicity from his AKI. His encephalopathy resolved after gabapentin discontinuation. 

The patient was discharged to home health care on hospital day 20, per patient preference, given his improved clinical picture. The patient was discharged on IV piperacillin-tazobactam 3.375 g in 0.9% NaCl q8h for a total duration of six weeks with scheduled follow-up with infectious disease in two weeks. The patient subsequently recovered from the infection and continued to endorse chronic knee pain in the lower left limb. However, interosseous infection was not evidenced on scans.

Overall, infectious disease expressed concern that his diverticulitis led to transient bacteremia that seeded in the left knee, given the absence of other risk factors such as the presence of hardware. Per infectious disease, this case was not one of antibiotic failure but rather failure of adequate source control.

Case 3

A 57-year-old male with a history of obesity, DM, chronic kidney disease (CKD), coronary artery disease (CAD), congestive heart failure (CHF), peripheral artery disease (PAD), and right below-knee amputation presented to the ED for left lower extremity pain. 

The patient's vital signs were unremarkable. The laboratory values showed leukocytosis, mild anemia, elevated creatinine, and elevated C-reactive protein (Table 2).

**Table 2:** Laboratory investigation of case 3.

Laboratory Name	Laboratory Value	Laboratory Reference Range
WBC	22,900 cells/µL	3,500-10,500 cells/µL
Hemoglobin	8.3 g/dL	13.5-17.5 g/dL
Creatinine	4.0 mg/dL	0.6-1.2 mg/dL
CRP	21.8 mg/dL	0.5-2.2 mmol/L

**Figure 5:**
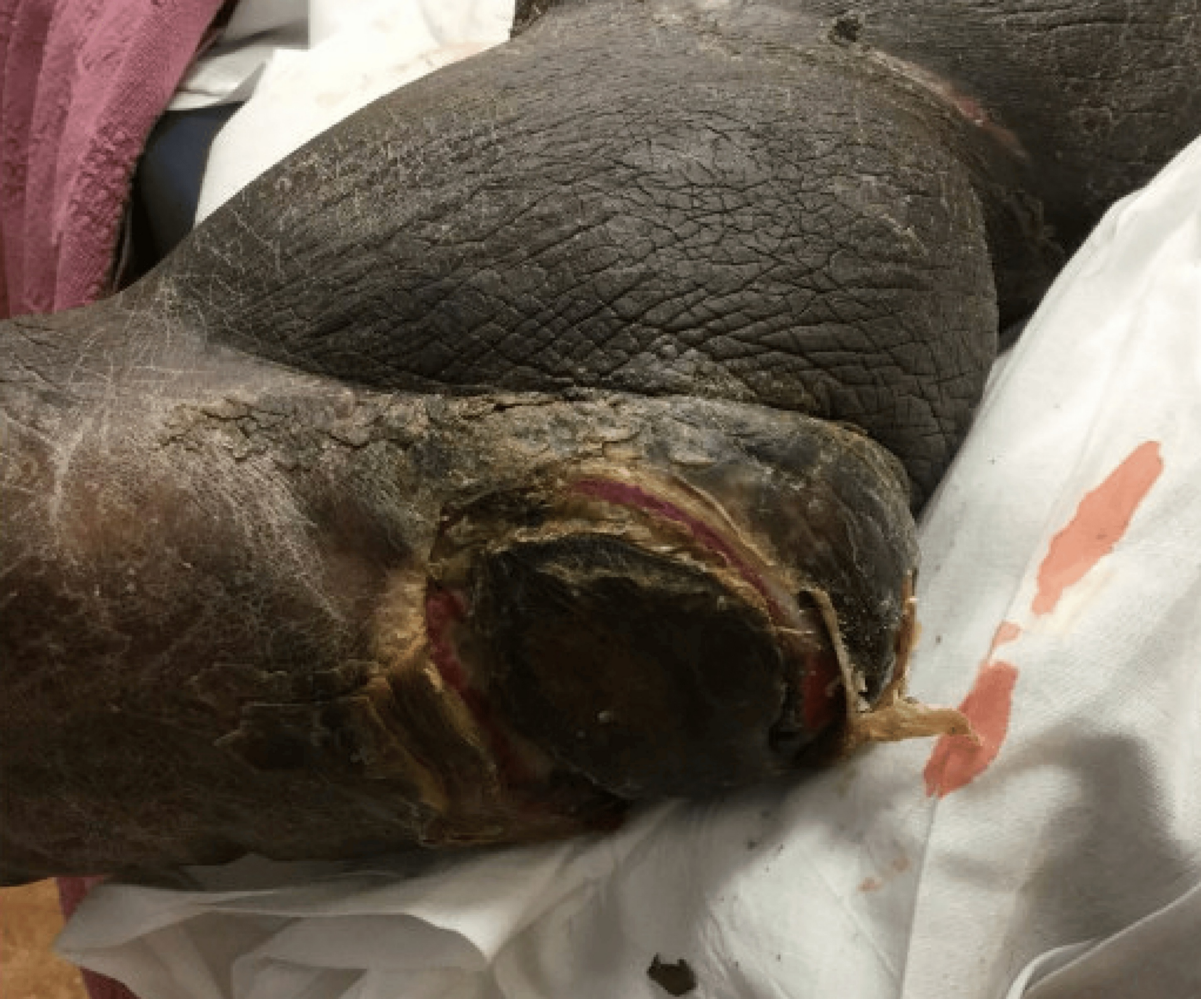
Open wound of the left calcaneus and sole of the foot.

Physical exam revealed a foul-smelling left foot with cellulitis and lymphedema. The skin was warm to touch. Gangrene with open wounds of the sole (2 cm in size) and the calcaneus (4 cm in size) were noted (Figure 5). The left calf and thigh were not tender to palpation.

Blood cultures × 2 were taken on admission to the hospital and demonstrated no growth by day 5. No wound cultures were taken during this hospital admission. Until the final result of the blood cultures was processed, the patient was administered IV piperacillin-tazobactam 3.375 g q8h. Of note, wound cultures three months prior to the lower left extremity grew Klebsiella pneumoniae and Proteus mirabilis, indicating a chronic infection due to type 2 DM. 

**Figure 6:**
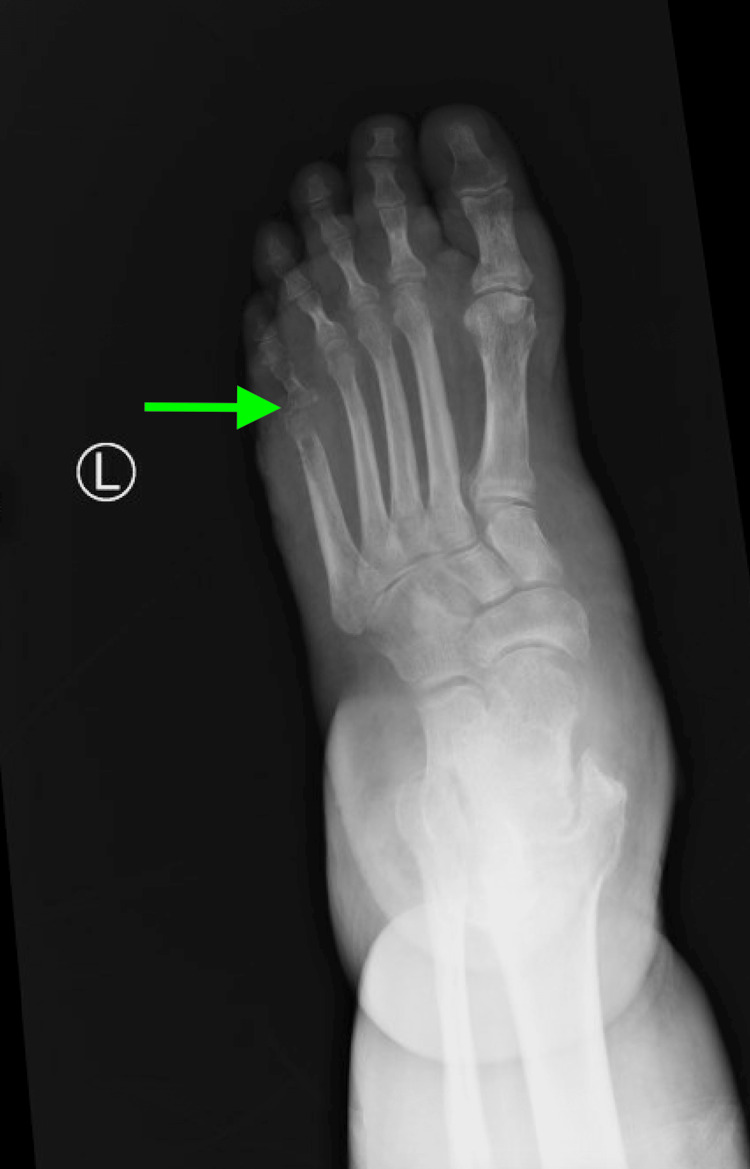
X-ray of the left foot.

An X-ray of the left foot revealed erosion and destruction of the distal fifth metatarsal and proximal aspect of the phalanx, with dorsomedial displacement of the proximal phalanx relative to the metatarsal bone at the joint. This was concerning for osteomyelitis with septic arthritis and possible fracture through the infected bone. Soft tissue swelling was noted diffusely (Figure 6).

**Figure 7:**
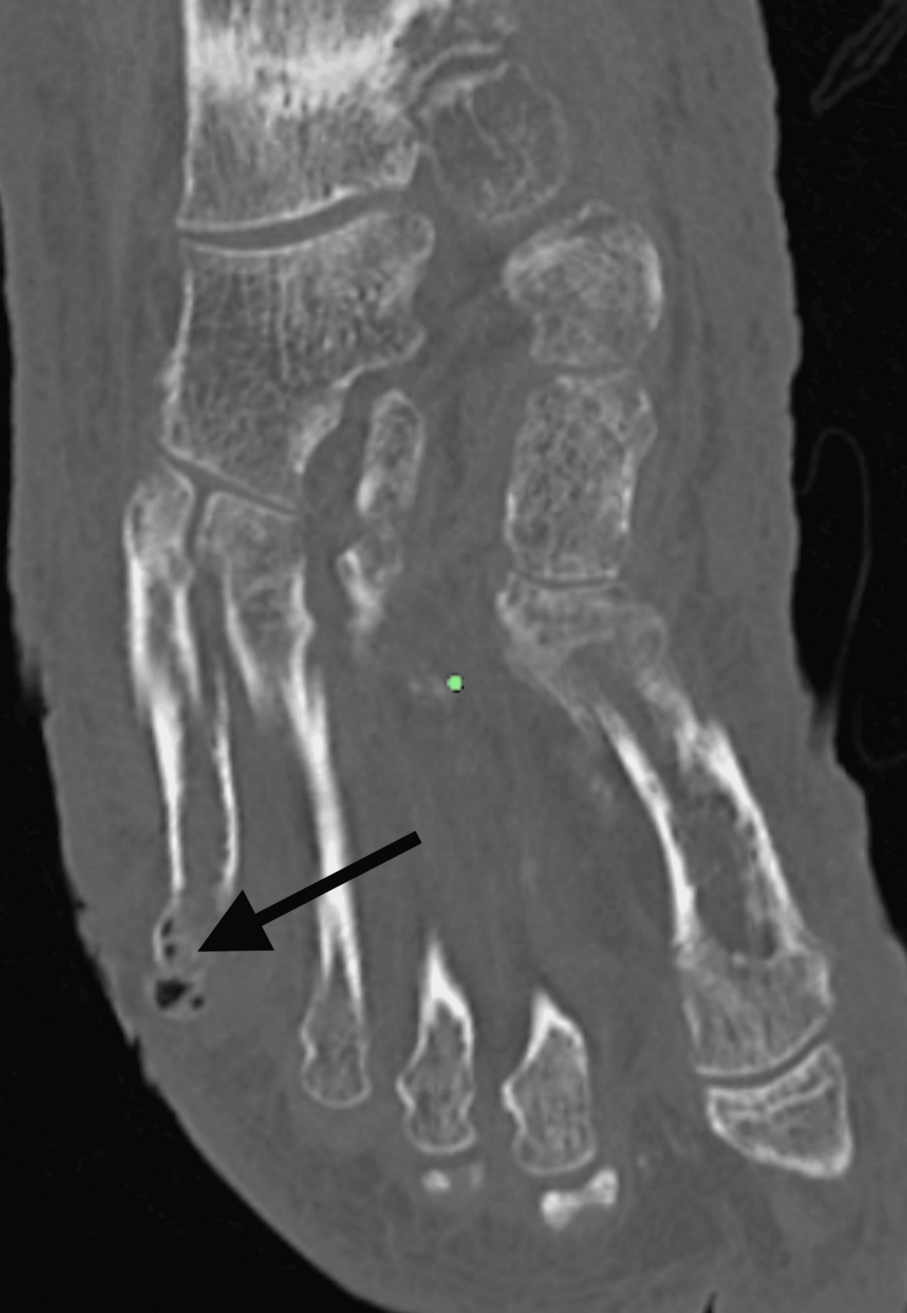
Emphysematous osteomyelitis of the left fifth metatarsal head. A CT sagittal view of the left foot in a 57-year-old man presenting with left lower extremity pain showing emphysematous osteomyelitis.

**Figure 8:**
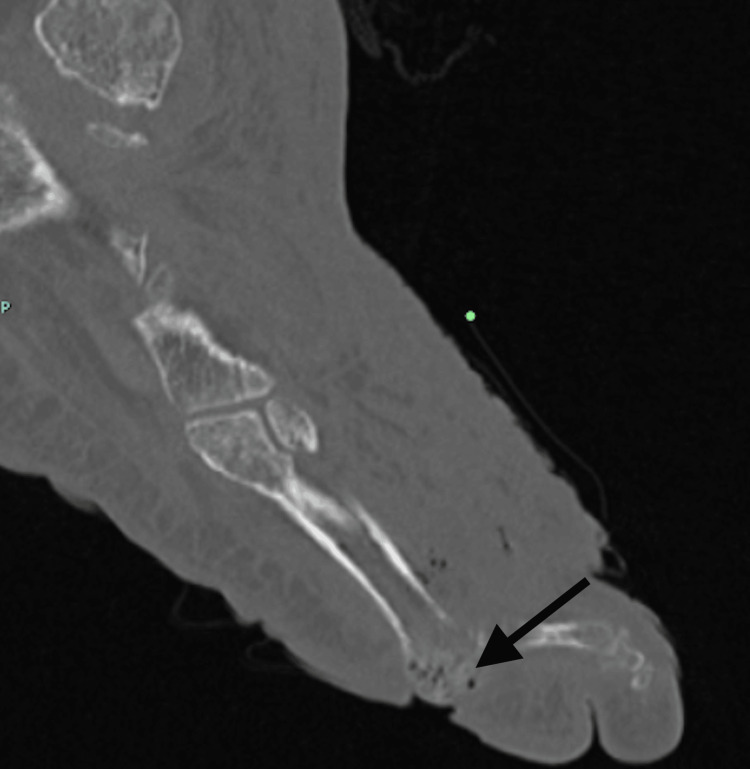
Emphysematous osteomyelitis of the left fifth metatarsal head. A CT of the left foot axial view in a 57-year-old man presenting with left lower extremity pain showing emphysematous osteomyelitis (black arrow).

A CT of the left foot revealed osteomyelitis of the fifth metatarsal head with an overlying soft tissue wound defect and metatarsophalangeal dislocation (Figure 7 and Figure 8).

An ankle disarticulation was performed on the day of admission. Additionally, a below-knee amputation was performed on hospital day 4. Pathology tissue results of the left lower extremity post-amputation revealed calcifying atherosclerosis and ulceration with viable soft tissue margins. Since blood cultures demonstrated no growth on hospital day 5, IV piperacillin-tazobactam was discontinued. The patient recovered from the surgery and infection and was discharged on hospital day 10. 

## Discussion

EO is a rare infection of the bone by gas-producing bacteria and is associated with a significant mortality rate of 32% [8]. EO has been shown to be more aggressive than general osteomyelitis, with fatality rates reported as high as 50% when the spine is affected, compared to just 10% from general osteomyelitis of the spine, since gas in the spine is often confused for other gas-producing conditions such as degenerative discs, osteonecrosis, and cancer [9].

The most common risk factor for EO is DM, with other risk factors including malignancy, radiotherapy, sickle cell disease, alcohol abuse, and Crohn’s disease [10]. The disease typically occurs in patients after trauma, biopsy, penetrating wounds, and fractures [4]. Bacteria from these sources are then able to travel to the bone via the bloodstream and often affect the pelvic bones (38%), vertebral bodies (32%), femur (24%), and others [10]. 

There are several bacteria identified as causative agents, many of which are members of the *Enterobacteriaceae* family [1]. Identified pathogens include *Pseudomonas*, *Enterococcus*, *Escherichia coli*, *Salmonella*, *Staphylococcus aureus*, *Streptococcus intermedius*, *Clostridium* spp., *Mycobacterium tuberculosis*, *Enterobacter*, and *Klebsiella* [1]. 

While both MRI and CT can be used, the gold standard for diagnosis is CT. Imaging for these patients has been described as irregular interosseous air bubbles giving a “pumice stone” sign that is created by the gas-producing microbes within the tissue [10]. Differential diagnosis for interosseous gas includes osteonecrosis, bone neoplasia, interventional procedures, penetrating wounds, compound fractures, and lymphangiomatosis of bone [8]. 

Treatment generally involves four to six weeks of IV antibiotic treatment based on the causative agent and/or surgical intervention, depending on the spread of infection [11].

## Conclusions

Case 1 is an extremely rare case demonstrating multifocal EO. The notable locations of EO included vertebral bodies T1, T8, T11, T12, and L1, the T5 spinous process, as well as the right iliac bone and right sacroiliac joint. Because of the multifocal nature and severity of the condition, the patient ultimately passed away, making early and accurate diagnosis critical. If EO is suspected, a CT should be ordered to confirm the presence of gas in the bone. Following the CT, an MRI should be performed to view the full extent of the disease. Cases 2 and 3 both feature a *Proteus* infection as well as a previous diagnosis of DM, a known risk factor for EO. Case 3 is a more classic case where diabetic neuropathy leads to an injury of the foot, causing local infection and then osteomyelitis.

EO is a rare but dangerous disease that can potentially be fatal if not diagnosed and treated quickly, emphasizing the importance of a thorough medical history and accurate radiography.
